# ASO Author Reflections: Characteristics of Locally Recurrent Rectal Cancer in the Lateral Compartment

**DOI:** 10.1245/s10434-026-19235-7

**Published:** 2026-02-07

**Authors:** F. E. C. Vande Kerckhove, D. M. J. Creemers, E. Banken, S. H. J. Ketelaers, R. R. J. Coebergh van den Braak, G. A. P. Nieuwenhuijzen, A. E. Verrijssen, A. W. Daniëls-Gooszen, T. R. van Oudheusden, S. G. van Ravensteijn, I. E. G. van Hellemond, H. J. T. Rutten, H. M. U. Peulen, J. G. Bloemen, J. Nederend, J. W. A Burger

**Affiliations:** 1 Department of Surgery, Catharina Hospital Eindhoven, Eindhoven, The Netherlands; 2 GROW Research Institute for Oncology and Reproduction, Maastricht University, Maastricht, The Netherlands; 3 Department of Medical Oncology, Catharina Hospital Eindhoven, Eindhoven,, The Netherlands; 4 Department of Radiation Oncology, Catharina Hospital Eindhoven, Eindhoven, The Netherlands; 5 Department of Radiology, Catharina Hospital Eindhoven, Eindhoven, The Netherlands

## Past

Locally recurrent rectal cancer (LRRC) involving the lateral pelvic compartment is associated with poorer overall survival and disease-free survival than are central recurrences.^1–3^ Despite this, the mechanisms driving lateral LRRC remain inadequately characterized. A more comprehensive understanding of the factors contributing to lateral LRRC is pivotal for the further optimization of treatment strategies. Accordingly, the aim of our study was to characterize the radiological features of lateral LRRC and assess their anatomical relationship to primary tumor features in a large retrospective cohort, following central radiological and expert panel review.

## Present

This study encompassed all patients diagnosed with lateral LRRC at a national tertiary referral center in the Netherlands between 2018 and 2025 (*n* = 104). Primary and recurrent tumor magnetic resonance images were centrally re-reviewed to evaluate tumor features. An expert panel further radiologically assessed the anatomical relationship between the primary tumor location and the subsequent site of lateral LRRC. The analyses revealed that the primary tumors of lateral LRRC frequently exhibited high-risk radiological features such as extramural vascular invasion (45%), cT3cd-cT4ab staging (47%), and tumor deposits (23%).^4^ A total of 80% of lateral LRRC had no enlarged lateral (pelvic) lymph nodes (LLN+) at the time of the primary tumor.^4^ Following expert panel review, less than half of LLN+ primary tumors were subsequently linked to LLN nodal lateral local based on radiological assessment.^4^ Overall, 11% of lateral LRRC could be attributed to LLN nodal recurrence visible on primary imaging. Conversely, the remaining 89% appeared to be anatomically related to primary tumor spread and other high-risk tumor features.

## Future

Our analysis supports the finding that lateral LRRC predominantly presents as multicompartmental disease and appears to arise from multifactorial etiology. Notably, only a minority of lateral LRRCs could be attributed to primary LLN+, suggesting that LLN metastases constitute just one of several pathways contributing to lateral LRRC. Furthermore, LLN metastases, along with other tumor characteristics such as extramural vascular invasion, advanced T-stage, and tumor deposits appear to reflect aggressive tumor biology. To better guide (neoadjuvant) treatment decisions, future studies focusing on the development and validation of risk models that incorporate tumor biology and anatomical spread are warranted.
